# Supplementary material for: Geospatial interpolation and hydro-geochemical characterization of alluvial aquifers in the Thal Desert, Punjab, Pakistan
Source: PLoS One. 2024 Aug 14;19(8):e0307025. doi: 10.1371/journal.pone.0307025 (PMC11324156; doi:10.1371/journal.pone.0307025)
Supplement: Attachment — Submitted filename: Respose to reviewers.docx [file pone.0307025.s007.docx]

The authors are very thankful to the editor and the reviewers for their valuable time and comments. We have revised the manuscript in the light of the suggestions and comments of the reviewers and the editor. Please see the “**Revised Manuscript (Marked up copy).docx**”. All important changes made in the manuscript are tracked using the 'Track Changes' in the manuscript file.

The response of each comment is given below in tabular form. We are hopeful that the manuscript is now ready for acceptance.

Kind regards,

Muhammad Irfan Ehsan (Ph.D.)

| **Sr. No.** | **Editor comments** | **Answers** |
| --- | --- | --- |
| 1 | Please amend the title either on the online submission form or in your title page so that they are identical | Thank you for bringing this to our attention. We have revised the title to ensure consistency between the online submission form and the title page of the revised manuscript. The title now matches on both the submission form and in the revised manuscript. |
| 2 | In your Methods section, please provide additional information regarding the permits you obtained for the work. Please ensure you have included the full name of the authority that approved the field site access and, if no permits were required, a brief statement explaining why. | We appreciate your attention to the permitting process for our work. In the case of our study, no specific permits were required as the research was conducted in a public area. Public properties typically do not necessitate permission from a specific authority for access. Therefore, we conducted our research in accordance with the rules and regulations governing public spaces, and no formal approval from any specific authority was needed. |
| 3 | Please note that PLOS ONE is unable to publish previously copyrighted maps or satellite images, or images created using proprietary data. For these reasons, we cannot publish images generated by software which copyrights their output. In order to use these images in your submission, we require explicit permission from the copyright owner to publish the figures under the CC BY 4.0 license.  At this time, please kindly clarify the following regarding Figures 1, 2, and 11 remaining in your submission:  a) Where did the authors obtain the maps, basemaps, shapefiles, map data, etc. in Figures 1, 2, and 11?  b) Please state whether the maps, basemaps, shapefiles, map data, etc. in these images have been previously copyrighted to your knowledge.  c) If any of the maps, basemaps, shapefiles, map data, etc. in these images have been previously copyrighted, we require specific consent from the copyright holder(s) to publish these images in PLOS ONE, under the CC BY 4.0 license. | In response to the inquiries regarding Figures 1, 2, and 11, we have made the following clarifications:  a) Fig 1: The maps in Figure 1 have been revised, and copyrighted maps have been replaced with self-generated maps that do not contain any copyrighted content.  b) Fig 2 & 11: Both Figures 2 and 11 depict self-generated maps created through GIS processing, specifically employing interpolation techniques. The physiochemical database used for interpolation and the shapefiles are all generated by our team. To the best of our knowledge, none of the maps, basemaps, shapefiles, or map data in these images have been previously copyrighted.  c) Given the revisions made to Fig 1 and the self-generated nature of Fig 2 and 11, there are no images with copyrighted content remaining in our submission. We confirm that all necessary data and maps used are original creations by our team. These figures are now suitable for publication under the CC BY 4.0 license. |

| **Sr. No.** | **Journal Requirements** | **Answers** |
| --- | --- | --- |
| 1 | Please ensure that your manuscript meets PLOS ONE's style requirements, including those for file naming. The PLOS ONE style templates can be found at  <https://journals.plos.org/plosone/s/file?id=wjVg/PLOSOne_formatting_sample_main_body.pdf> and  <https://journals.plos.org/plosone/s/file?id=ba62/PLOSOne_formatting_sample_title_authors_affiliations.pdf> | We have carefully revised our manuscript to comply with PLOS ONE's style requirements, including proper file naming. The manuscript now adheres to the formatting guidelines provided in the PLOS ONE style templates.  We appreciate your attention to detail and the resources you've provided. If there are any additional aspects that need further adjustment, please feel free to let us know. |
| 2 | In your Methods section, please provide additional information regarding the permits you obtained for the work. Please ensure you have included the full name of the authority that approved the field site access and, if no permits were required, a brief statement explaining why. | Thank you for your guidance. The Methods section has been updated to include information about the permits obtained. Field site access for this study was approved by the Institute of Geology, University of the Punjab. We have also implemented track changes to reflect these revisions in the manuscript. |
| 3 | PLOS requires an ORCID iD for the corresponding author in Editorial Manager on papers submitted after December 6th, 2016. Please ensure that you have an ORCID iD and that it is validated in Editorial Manager. To do this, go to ‘Update my Information’ (in the upper left-hand corner of the main menu), and click on the Fetch/Validate link next to the ORCID field. This will take you to the ORCID site and allow you to create a new iD or authenticate a pre-existing iD in Editorial Manager. Please see the following video for instructions on linking an ORCID iD to your Editorial Manager account: <https://www.youtube.com/watch?v=_xcclfuvtxQ> | Thank you for the reminder. We have updated the ORCID information for the corresponding author in Editorial Manager. The ORCID iD is now validated and linked to the Editorial Manager account. |
| 4 | We note that Figures 1,2 and 11 in your submission contain map/satellite images which may be copyrighted. All PLOS content is published under the Creative Commons Attribution License (CC BY 4.0), which means that the manuscript, images, and Supporting Information files will be freely available online, and any third party is permitted to access, download, copy, distribute, and use these materials in any way, even commercially, with proper attribution. For these reasons, we cannot publish previously copyrighted maps or satellite images created using proprietary data, such as Google software (Google Maps, Street View, and Earth). For more information, see our copyright guidelines: <http://journals.plos.org/plosone/s/licenses-and-copyright>.   We require you to either (a) present written permission from the copyright holder to publish these figures specifically under the CC BY 4.0 license, or (b) remove the figures from your submission:   a. You may seek permission from the original copyright holder of Figures 1,2 and 11 to publish the content specifically under the CC BY 4.0 license.     We recommend that you contact the original copyright holder with the Content Permission Form (<http://journals.plos.org/plosone/s/file?id=7c09/content-permission-form.pdf>) and the following text: “I request permission for the open-access journal PLOS ONE to publish XXX under the Creative Commons Attribution License (CCAL) CC BY 4.0 (<http://creativecommons.org/licenses/by/4.0/>). Please be aware that this license allows unrestricted use and distribution, even commercially, by third parties. Please reply and provide explicit written permission to publish XXX under a CC BY license and complete the attached form.”   Please upload the completed Content Permission Form or other proof of granted permissions as an "Other" file with your submission.   In the figure caption of the copyrighted figure, please include the following text: “Reprinted from [ref] under a CC BY license, with permission from [name of publisher], original copyright [original copyright year].”   b. If you are unable to obtain permission from the original copyright holder to publish these figures under the CC BY 4.0 license or if the copyright holder’s requirements are incompatible with the CC BY 4.0 license, please either i) remove the figure or ii) supply a replacement figure that complies with the CC BY 4.0 license. Please check copyright information on all replacement figures and update the figure caption with source information. If applicable, please specify in the figure caption text when a figure is similar but not identical to the original image and is therefore for illustrative purposes only. The following resources for replacing copyrighted map figures may be helpful:   USGS National Map Viewer (public domain): <http://viewer.nationalmap.gov/viewer/> The Gateway to Astronaut Photography of Earth (public domain): <http://eol.jsc.nasa.gov/sseop/clickmap/> Maps at the CIA (public domain): <https://www.cia.gov/library/publications/the-world-factbook/index.html> and <https://www.cia.gov/library/publications/cia-maps-publications/index.html> NASA Earth Observatory (public domain): <http://earthobservatory.nasa.gov/> Landsat: <http://landsat.visibleearth.nasa.gov/> USGS EROS (Earth Resources Observatory and Science (EROS) Center) (public domain): [http://eros.usgs.gov/#](http://eros.usgs.gov/) Natural Earth (public domain): <http://www.naturalearthdata.com/> | Thank you for bringing this to our attention. We have removed all copyrighted map/satellite images, and the current set of figures in the manuscript complies with the Creative Commons Attribution License (CC BY 4.0) as per the journal's guidelines. No copyrighted images remain in our submission.  We appreciate your guidance and the resources provided for replacing copyrighted map figures. If any further adjustments are needed, please let us know. |
| 5 | Please include captions for your Supporting Information files at the end of your manuscript, and update any in-text citations to match accordingly. Please see our Supporting Information guidelines for more information: <http://journals.plos.org/plosone/s/supporting-information>. | Thank you for your guidance. We have included captions for the Supporting Information files at the end of the manuscript. The changes have been made in accordance with the Supporting Information guidelines. |

| **Sr. No.** | **Comments for Reviewer #1** | **Answers** |
| --- | --- | --- |
| 1 | Title may reduced as if possible. | Thank you for your feedback. We have considered your suggestion and revised the title to "Geospatial Interpolation and Hydro-geochemical Characterization of Alluvial Aquifers in the Thal Desert, Punjab, Pakistan." We believe this modification aligns with your recommendation for a shorter title while retaining the essential focus of our study. |
| 2 | L226 suspects high amount of turbidity in groundwater, mostly it may be within limits. | Thank you for highlighting the concern regarding potential high turbidity. High turbidity in these samples may be due to well conditions, considering factors such as improper packing, inadequate sealing, lack of gravel pack, well interference, and maintenance lapses. |
| 3 | The complete physical chemical parameters of each sample, please indicated in separate table. | Thank you for your feedback. We would like to bring to your attention that a comprehensive table titled " **S1 File** " has been provided in the “Supporting Information” material. This table includes detailed information on each physical and chemical parameter for every sample. We hope this addresses your concern. If you have specific suggestions for improvement or if further clarification is needed, please let us know. |
| 4 | L141 incubation temperature may conformed, coliforms, ecoli were not discussed. | Thank you for your valuable feedback. We would like to inform you that the suggestion regarding the incubation temperature has been confirmed. Additionally, a comprehensive discussion on coliforms and E. coli has been included. You can review the track changes in the revised manuscript. |
| 5 | The results and discussion that did not referenced and studies may find correlation with global or local studies. | Thank you for your feedback. We thoroughly investigated the possibility of referencing local studies but unfortunately, there are no studies in close proximity that directly correlate with our research. The distinct geomorphological and hydrogeological conditions in our study area limit the applicability of regional or global studies. |
| 6 | The figures are of poor resolution 300dpi. | Thank you for your feedback. We have addressed the concern about figure resolution by enhancing the resolution upto 800 dpi. |
| 7 | The temporal and spatial an objective, that may explained well with the references. | We have carefully addressed your comment by providing detailed explanations with references regarding the spatial variations of major ions, trace elements, and physicochemical parameters in Results and in Table 1. The concentrations and spatial variations have been thoroughly evaluated. However, we acknowledge that temporal variations were not assessed, and necessary changes have been made in the manuscript. |
| 8 | The samples may classified as geogenic and anthropogenic. | Thank you for your insightful comment. While our current study did not specifically focus on classifying samples as geogenic or anthropogenic, we appreciate your suggestion. We are planning to conduct a comprehensive geophysical survey, including Electrical Resistivity Tomography for demarcating quality water zones, coupled with a hydrostratigraphic study using borehole data. With this additional information, we aim to classify the samples and groundwater zones into geogenic and anthropogenic categories. |

| **Sr. No.** | **Comments for Reviewer #2** | **Answers** |
| --- | --- | --- |
| 1 | Hydrogeology of the area should be provided in detail. | Thank you for your feedback. We have made significant revisions to the manuscript to include a detailed section on the hydrogeology of the study area. These changes are tracked for your convenience. We trust that the updated content fulfills the requirement for a comprehensive discussion on the hydrogeological aspects. |
| 2 | How the analytical quality maintained and validated. | We maintained analytical quality by ensuring proper preservation of groundwater samples for laboratory testing, and all analyses were conducted with meticulous care. Additionally, to validate our findings, we observed alarming health issues among residents, and their reviews provided valuable insights. To further address the issue comprehensively, we are planning to conduct a geophysical survey and health assessments. These endeavors aim to identify the presence of geogenic and anthropogenic contaminants and inform potential remediation strategies for the health challenges faced by the local community. |
| 3 | The discussion part is very poor, need to discuss results in detail with causes and effects. It is not clear from where the contaminants are added to groundwater. | We have carefully revised the discussion section to provide a detailed analysis of the results, including causes and effects of the observed contaminants in groundwater and about the sources of contaminants. Sources of contamination is also discussed in results section, specifically in the "Geospatial Analysis of Water Quality Parameters." Additionally, to further enhance our understanding and identify contamination sources definitively, we are planning to conduct a geophysical survey. The integration of this survey data with our current findings will contribute significantly to elucidating causes, effects, and contamination sources. We hope these enhancements address your concerns. |
| 4 | There are very few cited papers on the hydrogeology of Indus basin and groundwater status of the area, consult following articles and cite MacAllister, et al 2022. A century of groundwater accumulation in Pakistan and northwest India. Nature Geoscience. <https://doi.org/10.1038/s41561-022-00926-1> Bonsor et al 2017. Hydrogeological typologies of the Indo-Gangetic basin alluvial aquifer, South Asia. Hydrogeology Journal. 25(5): 1377-1406 DOI: 10.1007/s10040-017-1550-z | Thank you for your valuable recommendations. We have incorporated the suggested references, including MacAllister et al. (2022) and Bonsor et al. (2017), to enhance the discussion on the hydrogeology of the Indus Basin and groundwater status in the study area. Additionally, we have expanded the hydrogeological section with further references to provide a more comprehensive overview. We hope these revisions align with your expectations. |
