# Supplementary material for: Geospatial interpolation and hydro-geochemical characterization of alluvial aquifers in the Thal Desert, Punjab, Pakistan
Source: PLoS One. 2024 Aug 14;19(8):e0307025. doi: 10.1371/journal.pone.0307025 (PMC11324156; doi:10.1371/journal.pone.0307025)
Supplement: Attachment — Submitted filename: Respose to reviewer.docx [file pone.0307025.s008.docx]

The authors are very thankful to the editor and the reviewers for their valuable time and comments. We have revised the manuscript in the light of the suggestions and comments of the reviewers and the editor. Please see the “**Revised Manuscript (Marked up copy).docx**”. All important changes made in the manuscript are tracked using the 'Track Changes' in the manuscript file.

The response of each comment is given below in tabular form. We are hopeful that the manuscript is now ready for acceptance.

Kind regards,

Muhammad Irfan Ehsan (Ph.D.)

| **Sr. No.** | **Comments for Reviewer #1** | **Answers** |
| --- | --- | --- |
| 1 | The manuscript did not explained well and short. | We appreciate your review of our manuscript. Though, we have reviewed carefully the manuscript and diligently revised the Introduction for better clarity, augmented microbial analysis in results, and included a comparative study in the discussion section, providing a more robust framework. These changes contribute to a technically sound and comprehensive manuscript. We believe the improvements align with the journal's standards and your reconsideration. |
| 2 | The table of physicochemical parameters of all 40 samples may also provided with sampling names and GIS locations. | Thank you for your insightful feedback. We have addressed the concern raised regarding the physicochemical parameters table for all 40 samples. We have included a comprehensive Excel sheet in the supporting information that provides the names, GIS locations, and corresponding physical and chemical analysis values for each sample. This addition aims to enhance the accessibility and completeness of the dataset, ensuring a more thorough understanding of our study. |
| 3 | Introduction was short with less relevant information. | We have taken your suggestion and significantly improved the introduction by incorporating additional relevant information supported by appropriate references. Our aim is to provide a more comprehensive and contextually rich introduction that aligns with the standards of the journal. |
| 4 | The samples may explained with respect to spatial variation and hierarchal analysis. | We appreciate your thoughtful feedback. The spatial variation of samples has been thoroughly elucidated. There are different type of analysis are in practice to demonstrate spatial variation of samples like hierarchal analysis (HA). We do not think that in this study HA is important. Regarding hierarchical analysis, we acknowledge the importance of this approach. However, as part of our future research plans, we are intending to conduct geophysical and physiochemical study in other areas of Muzaffargarh. We plan to incorporate hierarchical analysis in that study, and in the interim, we will acquire the necessary skills and tools for its effective implementation. Your insightful suggestion has been duly noted, and we look forward to incorporating hierarchical analysis in our forthcoming research. |
| 5 | The microbial count may explained in groundwater as indicated Line 284, this may added as separate section. | Thank you for your insightful feedback. To address your suggestion regarding microbial count in groundwater (referenced in Line 284), we have created a distinct section for microbial analysis in both the methodology and results. This modification enhances the clarity and organization of our manuscript. We have diligently incorporated relevant content into these new sections, and track changes have been applied for your ease of review. Your input has been invaluable, and we trust that these adjustments contribute to a more cohesive and detailed presentation of our study. |
| 6 | The water dependency of total population and houses on each sample may also explained and may present in table. | We appreciate your valuable suggestion regarding the water dependency of the total population and houses for each sample. While acknowledging the importance of this aspect, we regret to inform you that conducting a survey for this consideration within the current scope is challenging due to time and budget constraints and does not has any relevancy with the topic of this study. |
| 7 | The ion balance error may also explained. | We appreciate your input on the ion balance error. In this study, our primary focus as geoscientists was the spatial distribution, geochemical analysis, and contamination sources in groundwater. Due to the current scope, we did not delve into explaining the ion balance error. |
| 8 | Point 2 not acceptable as high turbidity of most handpumps. | Thank you for your thorough review. Regarding the concern raised in Point 2, it's important to clarify that only four samples (S-10, S-20, S-21, S-37) exhibited unexpectedly high turbidity values (190.7, 53, 84, and 60 NTU). Our analysis suggests that these elevated readings may be attributed to factors such as shallower depth water extraction, the presence of organic matter, and specific well conditions. Conditions like improper packing, inadequate sealing, lack of gravel pack, well interference, and maintenance lapses could contribute to the observed high turbidity. |
| 9 | Point 5 did not responded by addition of references of relevant studies from local or abroad. Although literature present for water quality of Muzaffargarh that did not included here. | Thank you for your feedback on Point 5. We have addressed this concern by incorporating references from relevant local studies in the discussion section, specifically comparing our findings with a local study conducted in Muzaffargarh. The necessary changes have been made using track changes to highlight the additions. We appreciate your guidance in enhancing the scholarly context of our work and ensuring that relevant literature is adequately referenced. |
| 10 | Point 6 the resolution may improve if possible. | Thank you for your feedback. We have addressed the concern about figure resolution by enhancing the resolution upto 800 dpi. |
| 11 | Kindly explain the purpose of using following diagrams Durov, and Gibbs diagrams, scatter plots, stiff and Schoeller. | Thank you for highlighting the need for clarification regarding the purpose of utilizing diagrams such as Durov, Gibbs, scatter plots, Stiff, and Schoeller in our paper. These graphical representations are integral to our hydrogeochemical analysis and provide a comprehensive understanding of groundwater chemistry. The Durov diagram, for instance, aids in portraying the hydrogeochemical attributes of diverse samples by highlighting the proportion of major anions and cations present. The Gibbs diagram serves to relate geological formations to water chemistry, offering insights into the factors influencing groundwater chemistry. Stiff plots visually showcase the chemical makeup of water samples, identifying dominant geochemical processes affecting groundwater quality. Schoeller diagrams indicate the homogeneity of groundwater sources. Scatter plots and correlation matrices help identify relationships between physicochemical parameters, offering valuable insights. Each diagram plays a pivotal role in unraveling the complexities of groundwater hydrochemistry, enhancing our ability to make informed interpretations and contributing to effective water management strategies. |
| 12 | Please correct in conclusion, the arsenic remains lower mostly; but here it indicated higher than magnesium and  potassium. | Thank you for your feedback on the conclusion section. We acknowledge the noted discrepancy in arsenic levels being higher than magnesium and potassium, contrary to the general trend. It is essential to clarify that while arsenic typically remains lower, variations can occur due to specific environmental factors. The prevalence of unlined sanitation and the presence of microbial organisms contribute to microbial degradation of organic matter, enhancing the reduction of iron oxy-hydroxides and leading to the release of arsenic into groundwater. This insight underscores the complex and dynamic nature of environmental factors influencing groundwater chemistry. We appreciate your attention to detail and assure you that this clarification will be incorporated to enhance the accuracy of our conclusions. |
| 13 | The results were not compared with the local studies or international. | Thank you for your valuable feedback. We have taken your suggestion seriously and addressed this concern by incorporating references from relevant local studies in the discussion section. Specifically, we have compared our findings with a local study conducted in Muzaffargarh, providing a more robust scholarly context. These enhancements have been made using track changes to ensure transparency and highlight the additions. We appreciate your guidance in refining our manuscript and ensuring that our work is well-contextualized with pertinent literature. |
| 14 | It may also added the literature, similar from deserts for the present data for its comparisons. | Thank you for suggesting the inclusion of literature from similar desert regions for comparisons. We endeavored to find studies addressing comparable challenges, but the higher population density, prevalence of unlined sanitation, and unique geomorphological and hydrogeological conditions in our study area limit the relevance of global studies. However, we have extensively compared our findings with pertinent local studies in the discussion section, providing a comprehensive contextual analysis. Your feedback has guided our efforts to enhance the manuscript, and we appreciate your understanding of the distinct challenges inherent in our study area. |
| 15 | The figures presented that were not clear, particularly figure 2. | Thank you for highlighting concerns about figure clarity, especially Figure 2. We've improved the resolution to 1000 dpi to enhance visibility and understanding. |
| 16 | SAR for agriculture may also explained. | Thank you for pointing out the need to explain SAR for agriculture. Considering our densely populated desert study area with minimal agricultural land, we have predominantly applied SAR values in the Wilcox diagram to assess groundwater suitability. This approach is tailored to the specific characteristics of our region. Your feedback is appreciated, and we trust this clarification aligns with your expectations. |
| 17 | The experimental may explained well i.e microbiol analysis | Thank you for your insightful feedback. To address your suggestion we have created a distinct section for microbial analysis in both the methodology and results. This modification enhances the clarity and organization of our manuscript. We have diligently incorporated relevant content into these new sections, and track changes have been applied for your ease of review. Your input has been invaluable, and we trust that these adjustments contribute to a more cohesive and detailed presentation of our study. |
| 18 | The samples may explained on basis with cluster depending on depth, direction, desert or non desert etc, principal analysis may also applicable. | Thank you for suggesting a cluster-based explanation for the groundwater samples, considering factors such as depth, direction, and desert or non-desert locations, along with the application of principal analysis. In our current study, we've employed spatial and graphical visualization techniques to comprehensively analyze groundwater chemistry, leveraging the skills and knowledge at our disposal. |
| 19 | The Pearson correlation did not explained. | Thank you for your comment regarding the Pearson correlation. We want to assure you that we have provided a detailed explanation of the Pearson correlation in the results section of our manuscript. |
