# Supplementary material for: Geospatial interpolation and hydro-geochemical characterization of alluvial aquifers in the Thal Desert, Punjab, Pakistan
Source: PLoS One. 2024 Aug 14;19(8):e0307025. doi: 10.1371/journal.pone.0307025 (PMC11324156; doi:10.1371/journal.pone.0307025)
Supplement: Attachment — Submitted filename: Respose to reviewer.docx [file pone.0307025.s009.docx]

The authors are very thankful to the editor and the reviewers for their valuable time and comments. We have revised the manuscript in the light of the suggestions and comments of the reviewers and the editor. Please see the “**Revised Manuscript (Marked up copy).docx**”. All important changes made in the manuscript are tracked using the 'Track Changes' in the manuscript file.

The response of each comment is given below in tabular form. We are hopeful that the manuscript is now ready for acceptance.

Kind regards,

Muhammad Irfan Ehsan (Ph.D.)

| **Sr. No.** | **Comments for Editor #1** | **Answers** |
| --- | --- | --- |
| **1** | This study on groundwater hydrogeochemistry in the southern Thal Desert of Pakistan offers valuable insights,  yet it could benefit from minor improvements. While the study effectively aims to identify contamination  sources and assess their impact on groundwater and the ecosystem, there's room for clearer quantification of  pollutant levels from identified sources. Additionally, the explanation of weathering processes and ion  exchange effects could be more detailed. Providing more context for the results within existing literature  would enhance the study's significance. Furthermore, the relatively small sample size might limit the  generalizability of the findings, and the focus solely on certain groundwater sources may overlook other  potential contributors to contamination. Lastly, while the study categorizes water quality, it lacks detailed  justification for the chosen thresholds. Addressing these minor issues would strengthen the study's overall  impact and relevance. | Thank you for your constructive feedback on our manuscript. We have addressed several key points in the revision process to enhance the clarity and significance of our study. Specifically, we have provided a detailed explanation of weathering processes and ion exchange effects in the discussion section, spanning from lines 485 to 509. This addition aims to provide a comprehensive understanding of these factors as they relate to groundwater hydrogeochemistry in the southern Thal Desert of Pakistan.  Regarding the quantification of pollutant levels, we have improved our analysis to better quantify pollutant levels from identified sources. Additionally, we have provided more context for our results within the existing literature to enhance the study's significance.  We acknowledge the limitation of the relatively small sample size and have provided a clearer discussion on the generalizability of our findings.  We believe these revisions strengthen the manuscript and address the minor issues raised. |

| **Sr. No.** | **Comments for Reviewer #1** | **Answers** |
| --- | --- | --- |
| 1 | The manuscript is still short with less discussions and needs improvements and corrections throughout manuscript. | We have made further substantial improvements to the introduction and discussion sections, enhancing the manuscript’s depth and clarity. We believe these revisions effectively address your concerns and have strengthened our analysis and presentation. |
| 2 | The TDS may derived from electrical Conductivity by multiply the 0.64 on standard temperature. | We are unsure about your specific request or concern regarding this point. |
| 3 | Abnormally in many cases the Mg almost equal to Ca. | You may be right but in many studies we have noticed that Mg and Ca values differ to each other e.g. Maheswaran (1999); Rapant et al. (2022). In this study we have followed standard procedures for sampling and lab tests, and our results reflect the measured values of Mg and Ca in lab analysis.  Maheswaran R, Morris S, Falconer S, Grossinho A, Perry I, Wakefield J, Elliott P. Magnesium in drinking water supplies and mortality from acute myocardial infarction in north west England. Heart. 1999;82:455–60.  [Stanislav Rapant](javascript:;);  [Veronika Cvečková](javascript:;);  [Patrik Čermák](javascript:;)  Enrichment of drinking water with Ca and Mg by a fluidized bed recarbonization reactor: a case study of Devičie, Slovak Republic  J Water Health (2022) 20 (4): 630–640. |
| 4 | The turbidity as WHO guidelines not acceptable >5 NTU for waters. The closed unhindered systems lake in groundwater the turbidity are questionable, standard methods and sampling are highly desirable for the publications. | We agree with the reviewer however, we assured you that we have adhered to standard methods for both sampling and lab tests. The high turbidity values observed are indeed unusual for such systems, and we have addressed the possible causes of these values in detail in our previous revision. |
| 5 | The microbiology in groundwater may questionable and explained the reasons. | Generally, the microbial contamination of groundwater can happen when wells, septic tanks and offal holes are poorly constructed. Small communities using septic tanks are especially vulnerable to microbial contamination of their drinking water. In this study area, the microbial contamination may be mainly due to the above mentioned sources as the area is remote and less develop without any concept of concealed septic tanks and confined sewerage pipelines. This is very unfortunate that majority of the people do not know about this contamination. Please see the manuscript for further detailed explanation. |
| 6 | How the TDS upto 500mg/l indicated the taste of water unpleasant. | Though an elevated TDS level can affect the taste of groundwater, it is not usually harmful to human health. However, readings above 500 mg/l may not feel bitter taste and require further investigation for toxic particles and heavy metals present tinto water. Some other factors such as groundwater storage methods (e.g., in clay pots or "Matka") can also influence the taste. |
| 7 | The points 1 and 4 as the spatial variations that did not well explained. | We have addressed these concerns and improved the explanations in our previous revision. We believe the current version provides a clearer and more comprehensive discussion of these spatial variations. |
| 8 | The points 6,7, 8, 9, 13, 14, 18 did not well explained. | Thank you for your comments on points 6, 7, 8, 9, 13, 14, and 18. We have elaborated on these points to the best of our knowledge and provided detailed answers in the previous revision. We believe our current explanations address these concerns thoroughly. |
| 9 | The figures did not yet improved. | We have exported our figures at 1000 dpi to ensure high resolution. The apparent decrease in resolution may be due to the submission system. We are happy to provide high-resolution versions through an alternative method if needed. |
| 10 | The results discussion were not compared with local and the world studies. | We have addressed this concern in our last revision, specifically in the discussion section from lines 514 to 533. We have included comparisons with relevant local and global studies to provide context for our findings. |
| 11 | The general order of As (as this was ppb level) that did not corrected in conclusion. | Could you please clarify your concern or provide more specific guidance on what you would like us to address regarding the order of arsenic in parts per billion (ppb) levels? |
| 12 | The recommendations may reduce with importance of present work. | While we appreciate your comments, we believe our recommendations play a crucial role in raising awareness among local governments and communities about water quality concerns. Our goal is to ensure the well-being and safety of individuals by advocating for precautions and promoting awareness. |
| 13 | The samples may discussed with each other, rather in groups. | Thank you for your comment regarding the discussion of samples. We have addressed this by discussing individual samples, both in the spatial variation section and the discussion section. |
